# Meta-Analysis of Down Syndrome Cortical Development Reveals Underdeveloped State of the Science

**DOI:** 10.3389/fncel.2022.915272

**Published:** 2022-06-13

**Authors:** Kirstin A. Risgaard, Isabella A. Sorci, Sruti Mohan, Anita Bhattacharyya

**Affiliations:** ^1^Waisman Center, University of Wisconsin—Madison, Madison, WI, United States; ^2^Department of Cell and Regenerative Biology, School of Medicine and Pubic Health, University of Wisconsin—Madison, Madison, WI, United States

**Keywords:** neuron, neural development, neurogenesis, immunohistochemistry, intellectual disability

## Abstract

Neurodevelopmental impairment contributes to the hallmark cognitive disability in individuals with Down syndrome (DS, trisomy 21, T21). The appearance of cognitive deficits in infancy suggests that alterations emerge during the earliest stages of neural development and continue throughout the lifespan in DS. Neural correlates of intellectual and language function include cortical structures, specifically temporal and frontal lobes that are smaller in DS. Yet, despite increased understanding of the DS cognitive-behavioral phenotype in childhood, there is very little structural and histological information to help explain the deficits. Consequently, attempts to effectively design therapeutic targets or interventions are limited. We present a systematic review of published research on cortical development in DS that reveals a paucity of studies that rigorously identify cellular features that may underlie the gross morphological deficits of the developing DS brain. We assessed 115 published reports retrieved through PubMed and other sources and found that only 23 reported histological and/or immunohistochemical data to define cell composition affected in DS post-mortem brain. Further, our analysis reveals that many reports have limited samples sizes and few DS samples, making it difficult to draw conclusions that are generally applicable to the DS population. Thus, the lack of replication and limited number of studies indicate that more developmentally focused research, ideally using equal numbers of age-matched samples in analyses, is needed to elucidate the cellular nature of smaller brain size in DS.

## Introduction

Neurodevelopmental impairment and neuronal dysfunction contribute to the hallmark cognitive disability in individuals with Down syndrome (DS, trisomy 21, T21). Little is known about what, when, and how specific neurons are affected that lead to intellectual disability. Consequently, attempts to effectively design therapeutic targets or interventions are limited. The cognitive phenotype of individuals with DS includes specific deficits in cognition, attention, working memory, motor development and language that begin in the first months of life and progress to have significant consequences on long-term academic, occupational, and daily life outcomes (Silverman, 2007; Frank and Esbensen, 2015; Faught et al., 2016; Esbensen et al., 2017). The appearance of these deficits in infancy suggests that alterations emerge during the earliest stages of neuronal specification, synaptogenesis and synaptic transmission in DS. Neural correlates of intellectual and language function include cortical structures, specifically temporal and frontal lobes, that are smaller in DS (Jung and Haier, 2007; Menghini et al., 2011; Carducci et al., 2013; Friederici and Gierhan, 2013; Van Den Heuvel and Sporns, 2013). Yet, there is very little cellular information of the prenatal period in DS to help explain the deficits (Hamner et al., 2018). Further, while recent neuroimaging studies provide information about size/volume and gross structure (Baburamani et al., 2019, 2020; Patkee et al., 2020; Tarui et al., 2020), they lack the resolution to provide insight into specific cortical neuron disruptions.

Minimal information is available about which neuronal subtypes are reduced and when during development they are reduced thus hindering their impact to our understanding of brain structure in DS. We carried out a systematic review of published research on cortical development in DS to identify publications that provide information on differences in cell numbers or cell structures that may inform the gross difference in brain structure of the cortex in DS. We focused on studies that provided histological and/or immunohistochemical data to define neuronal subtypes (e.g., neurons with distinct morphologies, in distinct locations or expressing specific markers) that are different in DS. Our analysis reveals a paucity of studies that rigorously assess cell types in the developing DS brain.

## Methods

A literature review was carried out in September 2020 and again in March 2022 to include new publications from 2020 to 2021 using PubMed and the search terms (Down syndrome) and (cortical development) and (human). We used the Preferred Reporting Items for Systematic reviews and Meta-Analyses (PRISMA) method to guide the design and reporting of the results (Moher et al., 2009; Page et al., 2021). Additional records were also identified through other sources.

Full text articles were assessed for eligibility (Supplementary Table 1) and studies were included in analysis (Supplementary Table 2). Publications were included in analysis if they provided information to indicate what, when and how specific neurons are affected in the DS cortex. Thus, inclusion criteria included (1) post-mortem human tissue, (2) comparison of control and DS, and (3) quantitative or qualitative information about neurons. Exclusion criteria: (1) analysis of cells *in vitro*, (2) study limited to expression (e.g., protein, RNA) differences with no parallel cellular information, (3) no histological staining or immunohistochemistry (e.g., MRI), (4) analysis of solely mouse models, (5) reviews or protocols that did not provide new data, and (6) studies not focused on DS specifically (e.g., intellectual disability).

## Results

**Figure 1:**
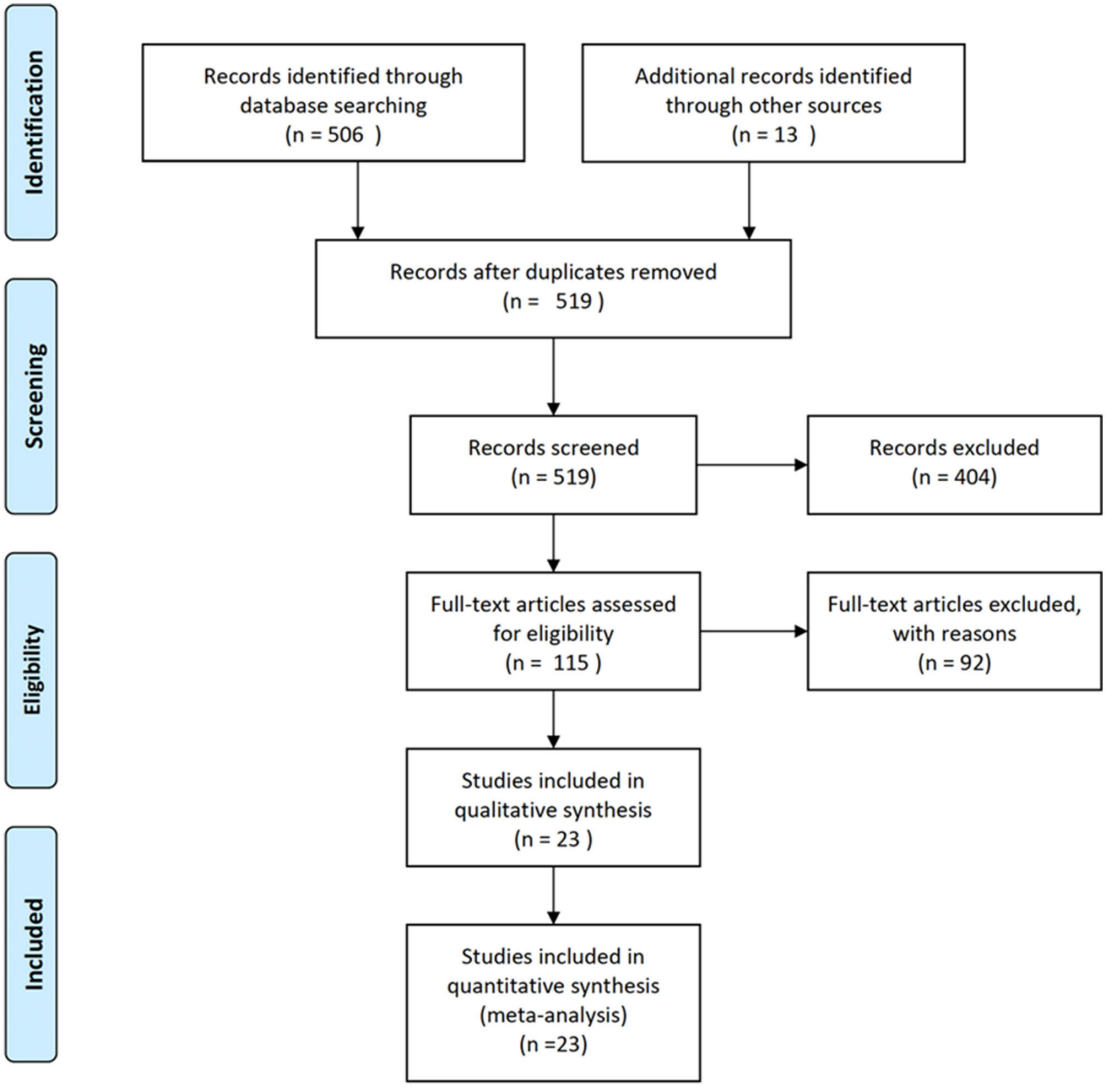
PRISMA flow diagram of the studies retrieved for the review.

Figure 1 shows the PRISMA flow diagram of the studies retrieved for the review. An initial 519 records were screened by title and over 400 were excluded because the title indicated that they were not relevant to this analysis. The remaining 115 full text articles were assessed for eligibility and, based on exclusion criteria, 23 studies were included in the meta-analysis, representing <5% of the initial identified records.

### Excluded Studies

More than 80% of the full text articles that were assessed did not meet our eligibility criteria (Figure 1; Supplementary Table 1). The most common reasons for exclusion were that studies were limited to expression (22%) or had no histological staining or immunohistochemistry (21%). Some of these excluded studies include valuable neuroimaging results that provide information about gross structure of the DS brain. Publications that focus on comparisons of expression levels of RNA and/or proteins in tissue may or may not take into account the structural or neuron subtype differences between DS and control tissue. Use of DS models, including cells (primary, stem cell derived, and/or non-neural) and mice, make up almost 20% of excluded studies. Excluded studies that do not specifically assess DS (9%), but do include intellectual disabilities may provide valuable information relevant to DS. Finally, 10% of the excluded studies were reviews of the literature and did not provide new data.

### Publication Date

**Figure 2:**
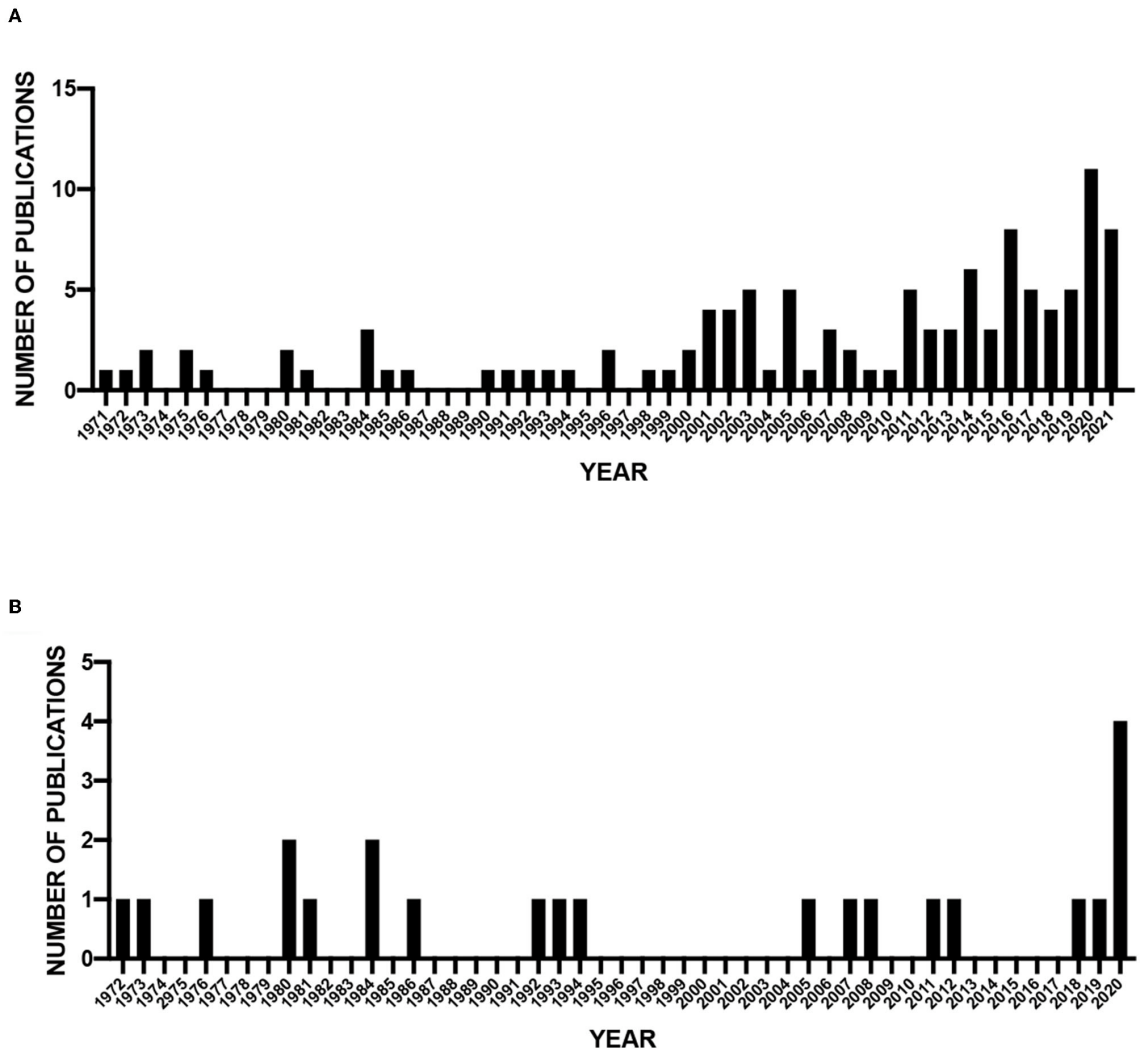
Publication dates of screened and analyzed publications. **(A)** Number of screened publications per year (1971–2021). **(B)** Number of publications included in meta-analysis (1972–2020).

While the number of studies that were included in this meta-analysis was <20% of the initial full-text articles assessed, we are able to identify interesting features in the small number of publications (Supplementary Table 2). First, we assessed publication dates of all articles (115) as well as those included in the meta-analysis (23) (Figure 2). There is a clear increase in the number of publications over time (Figure 2A). In contrast, the number of included publications that focus on cellular composition in the brain of DS individuals does not increase until very recently (Figure 2B). In addition, most years have only single publications and many years without publications. For example, no included studies were published between 1994 and 2005. These results indicate that while research in DS has increased in the last 20 years, there has not been a concomitant increase in our understanding of cellular differences in DS cortical development.

### Sample Size

**Figure 3:**
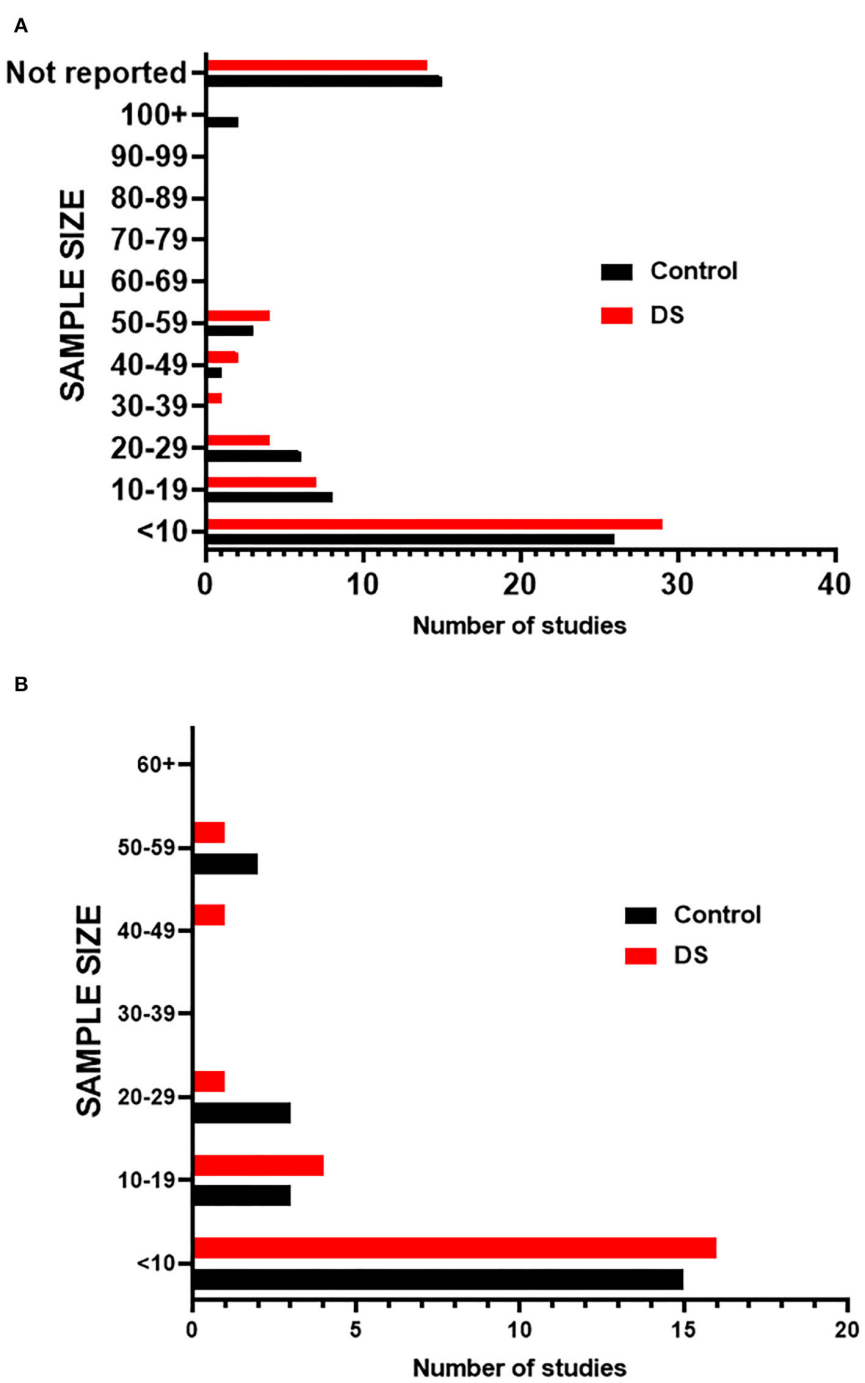
Sample sizes of studies. **(A)** Graph indicates the number of studies that fall within a given sample size range for both Control and DS samples in screened publications. **(B)** Graph indicates the number of studies that fall within a given sample size range for both Control and DS samples in publications included in meta-analysis.

We next assessed the studies for sample sizes that were given for both control and DS tissue (Supplementary Table 2). The results show that most studies evaluated few samples (<10) and a large proportion of the studies did not report samples size (Figure 3). Although similar numbers of samples were generally used for control and DS, the number of samples was often not matched. These small samples sizes may limit the ability to draw conclusions from these studies that are generally applicable to the DS population.

### Age of Samples

**Figure 4:**
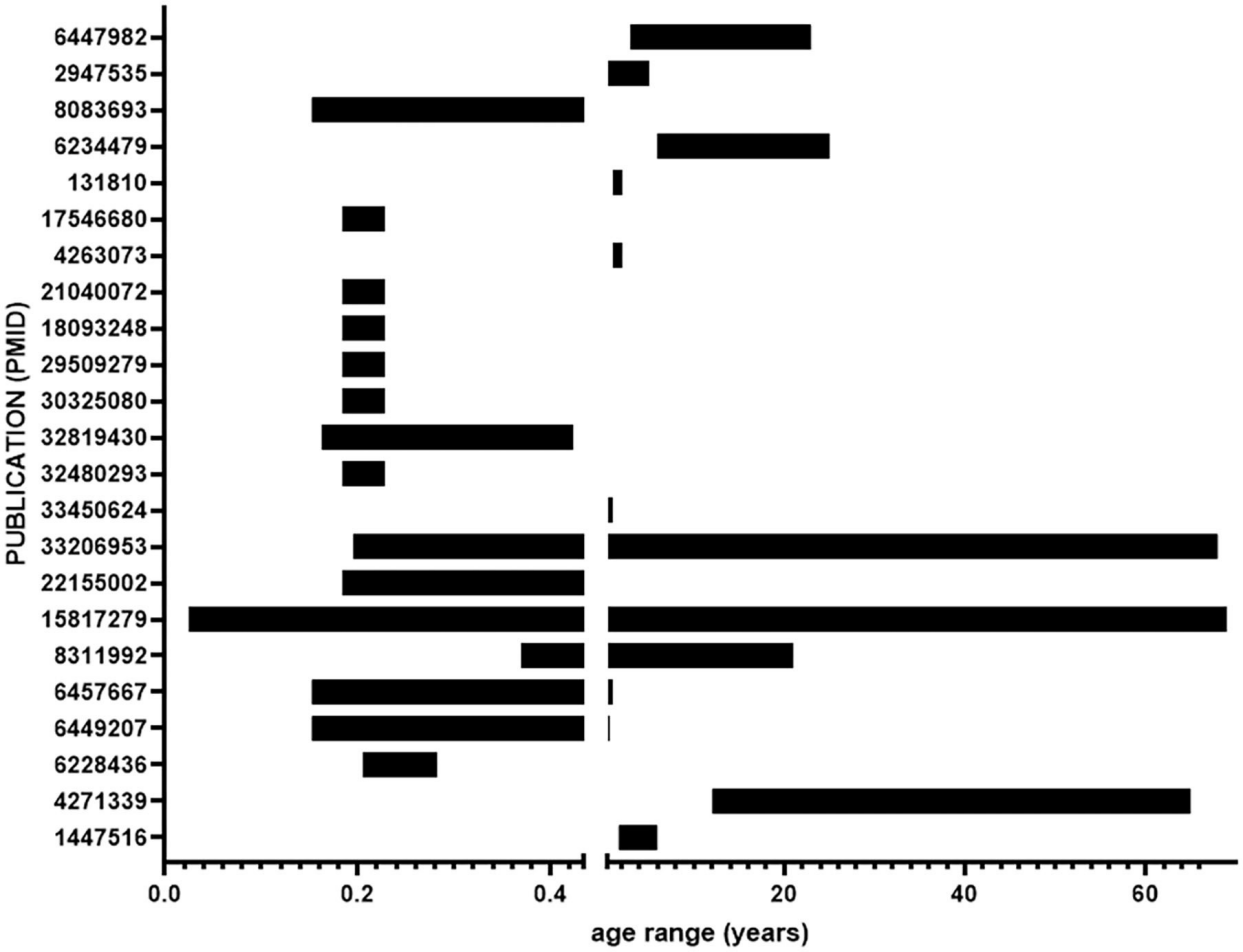
Age ranges of samples from included studies. Ages range from 16 days gestation to 69 years. The break in the *x*-axis indicates birth.

The impetus for carrying out this meta-analysis was to gain a better understanding of brain development in DS that necessitates prenatal and early postnatal tissue analysis. Many of the included studies analyzed tissue from a wide variety of ages (Figure 4), diverging from the goal of this meta-analysis. Factors such as environment and other age-related processes that naturally occur later in life make it difficult to synthesize results relevant to prenatal and early postnatal brain development from adult tissue samples. The large age ranges coupled with small sample sizes further erode the generalizability of the results.

### Synthesis of Findings From Analyzed Studies

Data based on small number of studies or small sample sizes are less reliable than data from rigorous and well-reproduced studies. Despite the limitations of the studies in this meta-analysis, results from all of the studies support a reduced volume and reduced neural cell numbers in brains of individuals with DS. It is possible that because cortical maldevelopment in DS is robust and highly penetrant, similar results are obtained from the limited studies. Yet, there remains little structural and histological information to help explain the deficits. Given that individuals with DS have neurodegeneration and Alzheimer's pathology in middle age, separating altered neurodevelopment from neurodegeneration requires separating analysis of prenatal and early postnatal ages from adult.

## Discussion

Our analysis revealed a limited number of studies that inform our understanding of brain structure in DS. Minimal information about which neuronal subtypes are reduced and when during development they are reduced is available, thus hindering their impact to our understanding of brain structure in DS. Our analysis revealed several factors that contribute to the limited impact of the studies: few studies, small samples size, and unmatched controls. The results point to the need for research focused on the prenatal and early postnatal cortical development to elucidate the cellular nature of smaller brain size in DS.

### Limitations of Search and Analysis

There are methodological limitations of our analysis including the chosen search terms and the limitation of search terms to capture known references. We needed to add known publications to supplement those retrieved from PubMed (Figure 1). Historical or non-indexed publications not retrieved by PubMed may have been omitted. We added several references from other sources that made up the majority of studies included in synthesis, highlighting the limitation of our search terms (Marin-Padilla, 1972, 1976; Suetsugu and Mehraein, 1980; Ross et al., 1984; Becker et al., 1993; Golden and Hyman, 1994; Contestabile et al., 2007; Guidi et al., 2008, 2011, 2018; Stagni et al., 2018, 2019, 2020). Despite the limitations of the search criteria and methodology, it is unlikely that we missed a significant number of publications that would change the outcome of our assessments or conclusions.

### Limited Information

Inaccessibility to human brain, especially prenatal brain, from individuals with DS necessitates the use of animal models, primarily mouse models of DS. Animal models have revolutionized biomedical research and facilitated the incredible advancement in our understanding of how the brain works. However, the utility of mouse models to define the neurodevelopmental missteps that occur in DS individuals is limited. Triplication of all human chromosome 21 genes and preservation of gene regulation is not possible in the mouse. Developmental processes in cortical formation that are likely defective in DS may be significantly different between human and mouse and so a more thorough and systematic inventory of neurons as well as dissection of underlying molecular machineries in DS brain and in human-relevant models is needed.

The effect of these limitations is exemplified by the predominant candidate mechanism underlying intellectual disability in DS that has emerged from research on mouse models—an imbalance in excitation-inhibition in the cortex, specifically over-inhibition due to over production of inhibitory GABA neurons and synapses (Best et al., 2007; Fernandez and Garner, 2007; Kleschevnikov et al., 2012a,b; Martinez-Cue et al., 2013, 2014; Potier et al., 2014; Zorrilla De San Martin et al., 2018). Whether over-inhibition occurs in DS human cortex remains unknown because no studies, as far as our analysis has shown, have systematically identified similar over production of inhibitory GABA neurons and synapses in humans with DS. A few studies have revealed a difference in ratios of excitatory to inhibitory neurons in DS, indicating fewer inhibitory neurons are present (Stagni et al., 2018, 2020) and only recently have excitatory-inhibitory synapse ratios been assessed in a single DS post-mortem sample (Sarnat and Flores-Sarnat, 2021). Additional studies focused on defining numbers of subtype-specific neurons (e.g., excitatory, inhibitory) and transmitter specific synapses (e.g., glutamate, GABA) in DS brain, and potentially human stem cell models, are needed to support or refute the over-inhibition hypothesis.

Our analysis reveals that many reports have limited samples sizes and few DS samples, making it difficult to draw conclusions that are generally applicable to the DS population. Thus, the lack of replication and limited number of studies indicate that more developmentally focused research on the prenatal and early postnatal period, ideally using equal numbers of age-matched samples in analyses, is needed to elucidate the cellular nature of smaller brain size in DS.

Further, the methods of analysis are often not rigorous. For example, the use of stereology takes into account the gross size differences of DS brains and ensures unbiased, efficient, and more reliable results than other *ad hoc* quantitative analyses (Gundersen et al., 1988; West, 1999, 2013; Perl et al., 2000; Boyce et al., 2010). Few studies take advantage of this rigorous methodology.

### Summary

Our meta-analysis of publications related to cortical development in individuals with DS revealed few studies that provide information on cellular composition in post-mortem brain. Further, our analysis reveals that many reports have limited samples sizes and few DS samples, making it difficult to draw conclusions that are generalizable.

Golgi and Nissl staining were used to label neurons in most of the analyzed reports. Studies using Golgi primarily assessed neuronal morphology and provide evidence for decreased synapses in DS brain (Marin-Padilla, 1972, 1976; Suetsugu and Mehraein, 1980; Takashima et al., 1981; Ross et al., 1984; Becker et al., 1986, 1993). However, the results are inconsistent, due largely to the broad age range of tissue examined. Thus, it is difficult to distinguish altered synaptogenesis from synapse loss due to neurodegeneration. Cell number and density were assessed either with Golgi, Nissl staining, or a combination of Nissl and immunocytochemistry and provide the best evidence for reduced numbers of neurons, and reduced neurogenesis in DS cortex underlying smaller volume (Golden and Hyman, 1994; Guidi et al., 2008, 2011, 2018; Contestabile et al., 2009; Stagni et al., 2018).

Expression data, especially of synaptic proteins, could be informative, but we chose to exclude these studies because without cell number/neuron information, interpretation is difficult. For example, less expression could be interpreted differently if there are the same numbers/types of neurons or, more likely, if there are fewer/different in DS.

Comprehensive datasets that define neuron composition in DS during cortical development are needed. Such datasets will serve as a reference for model systems, including animals or stem cells or other novel models. Validation of animal and human stem cell models through comparison of cellular and molecular phenotypes with *in vivo* data from individuals with DS is essential for studying DS pathogenesis. In particular, stem cell derived cortical organoids have been shown to display features of fetal cortical development (Pasca et al., 2015; Bershteyn et al., 2017; Di Lullo and Kriegstein, 2017; Pasca, 2018) and can thus be useful for identifying dysregulated developmental pathways in trisomy 21(Xu et al., 2019). Once validated, these models can expand our understanding of cortical development in DS. A complete understanding of cortical development is a critical first step toward identifying the biological mechanisms involved in cognitive and behavioral outcomes in DS to facilitate the development of targeted therapies and early interventions.

## Data Availability Statement

The original contributions presented in the study are included in the article/Supplementary Material, further inquiries can be directed to the corresponding author/s.

## Author Contributions

KR and IS contributed data, performed analysis, and edited paper. SM contributed data and performed analysis. AB conceived and designed analysis, collected data, contributed to analysis, wrote the paper, and secured funding. All authors contributed to the article and approved the submitted version.

## Funding

This work was supported by NIH grants (R03HD083538 and 1R01HD106197), the Jerome LeJeune Foundation, Wisconsin Partnership Program New Investigator Program, University of Wisconsin Alzheimer's Disease Research Center REC Scholar Award, UW-Madison and the Wisconsin Alumni Research Foundation and in part by a core grant to the Waisman Center from the National Institute of Child Health and Human Development (U54 HD090256).

## Conflict of Interest

The authors declare that the research was conducted in the absence of any commercial or financial relationships that could be construed as a potential conflict of interest.

## Publisher's Note

All claims expressed in this article are solely those of the authors and do not necessarily represent those of their affiliated organizations, or those of the publisher, the editors and the reviewers. Any product that may be evaluated in this article, or claim that may be made by its manufacturer, is not guaranteed or endorsed by the publisher.
